# Gene model for the ortholog of *GstO3* in *Drosophila immigrans*

**DOI:** 10.17912/micropub.biology.002238

**Published:** 2026-07-06

**Authors:** Jadon Bumgarner, Pablo Chialvo, Clare Scott Chialvo

**Affiliations:** 1 Biology, Appalachian State University, Boone, North Carolina USA

## Abstract

We developed a gene model for the *Glutathione S transferase O3* ortholog ( *GstO3* ) in the ASM1815216v1 Genome Assembly (GenBank Accession: GCA_018152165.1) of *Drosophila immigrans* . This ortholog was characterized as part of a developing dataset for a comparative study of detoxification gene family evolution in the *immigrans* - *tripunctata* radiation of the genus *Drosophila* using an adapted Genomics Education Partnership gene annotation protocol for Course-based Undergraduate Research Experiences.

**Figure 1. Genomic neighborhood and gene model for GstO3 in D. immigrans:** (A) **Synteny comparison of the genomic neighborhoods for *GstO3* in *Drosophila melanogaster* and *D. immigrans* .** Thin underlying arrows indicate which DNA strand the target gene, *GstO3* , is located on in *D. melanogaster* (top) and *D. immigrans* (bottom). The thin arrow pointing to the right indicate that *GstO3* is on the positive strand in *D. melanogaster* , and the thin arrow pointing to the left indicates that *GstO3* is on the negative strand in *D. immigrans* . The wide gene arrows pointing in the same direction as *GstO3* are on the same strand relative to the thin underlying arrows, while wide gene arrows pointing in the opposite direction of *GstO3* are on the opposite strand relative to the thin underlying arrows. White gene arrows in *D. immigrans* indicate orthology to the corresponding gene in *D. melanogaster* . Gene symbols given in the *D. immigrans* gene arrows indicate the orthologous gene in *D. melanogaster* , while the locus identifiers are specific to *D. immigrans* . (B) **Gene Model in GEP UCSC Track Data Hub** (Raney et al., 2014). The coding-regions of *GstO3* in *D. immigrans* are displayed in the User Supplied Track (red); coding sequences (CDS) are depicted by thick rectangles and introns by thin lines with arrows indicating the direction of transcription. Subsequent evidence tracks include Spaln of *D. melanogaster* Proteins (purple, alignment of Ref-Seq proteins from *D. melanogaster* ), Coding Regions Predicted by Augustus (dark blue), GeMoMa (teal), and NSCAN PASA-EST (dark green), and RNA-Seq from mixed sex adult flies (brown; alignment of Illumina RNA-Seq reads from *D. immigrans* – Erlenbach et al., 2023). (C) **Dot Plot of GstO3-PA in *D. melanogaster* ( *x* -axis) vs. the orthologous peptide in *D. immigrans* ( *y* -axis).** Amino acid number is indicated along the left and bottom; CDS number is indicated along the top and right, and CDSs are also highlighted with alternating colors. Line breaks in the dot plot indicate areas with low sequence similarity between species. A break extends over most of CDS 1 (purple box – a). In CDS 2, there are four small breaks and two longer breaks (dark green box – b; light blue box – c). (D) **Idiosyncrasies in protein alignment.** The break that extends over CDS 1 (purple box – a) is due to changes in amino acid sequence (one with high chemical similarity and a second with weakly similar chemical properties. CDS 2 contains two longer breaks in the protein alignment that indicate low levels of sequence similarity. In the first long break (dark green box – b), 21 of the 32 amino acids are conserved or highly chemically similar and seven are very dissimilar. The second long break (light blue box – b), covers 30 amino acids with 19 conserved or highly chemically similar and six very dissimilar. The four smaller breaks are associated with changes in one or two consecutive amino acids.

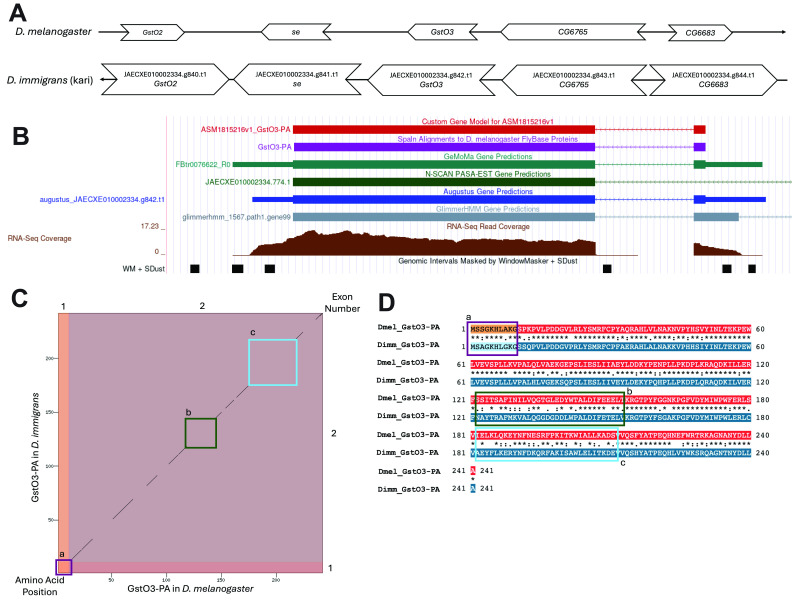



## Description

**Table:** 

* This article reports a predicted gene model generated by undergraduate work using a structured gene model annotation protocol defined by the Genomics Education Partnership (GEP; thegep.org ) for Course-based Undergraduate Research Experience (CURE). The following information in this box may be repeated in other articles submitted by participants using the same GEP CURE protocol for annotating Drosophila species orthologs of Drosophila melanogaster detoxification genes. * “Within insects, the process of detoxifying xenobiotics and host secondary metabolites is a three-phase process that involves functionalization, conjugation, and excretion of these compounds. Expansions of known detoxification gene families ( *e.g.* , cytochrome P450s) is associated with diet breadth and insecticide resistance (Ranson et al., 2002; Després et al., 2007; Rane et al., 2016). With the increasing availability of high-quality genomes for non-model organisms, including *Drosophila* species beyond *D. melanogaster* , it is now possible to perform large scale comparative studies (Robinson et al., 2011; Kim et al., 2021; Threfall and Baxter, 2021). Careful manual annotation and curation of gene models can improve upon computational gene predictions in non-model species, which aids the accuracy of studies on gene and genome evolution (Mudge and Harrow, 2016; Tello-Ruiz et al., 2019). To aid in these annotations, the Genomics Education Partnership (thegep.org) developed a curriculum involving web-based tools that allow undergraduates to engage in authentic course-based research focused on manually annotating genes in non-model species (Rele et al., 2023). The orthologous gene models, including the one presented here, then provide a reliable basis for further evolutionary genomic analyses when made available to the scientific community. The gene ortholog described here in *D. immigrans* for *Glutathione S transferase O3* ( *GstO3* ), a member of the glutathione S transferases (GSTs), was characterized as part of a developing dataset for a comparative study of detoxification gene families in the *immigrans* - *tripunctata* radiation of the genus *Drosophila* .” (Williams et al., 2026) Within the subgenus *Drosophila, D. immigrans* Sturtevant 1921 is placed in the *immigrans* subgroup of the *immigrans* species group within the *immigrans-tripunctata* radiation (Bächli, 2005). This cosmopolitan species is a generalist that feeds and develops on a variety of fruit (Sturtevant, 1921; Atkinson and Shorrocks, 1977; Atkinson, 1979). Unlike some other members of the radiation, *D. immigrans* does not tolerate the mushroom toxin α-amanitin (Jaenike et al., 1983). “One class of phase II detoxification enzymes are the glutathione S-transferases (GSTs), which act by conjugating xenobiotics or products of phase I detoxification with a glutathione to make them more hydrophilic prior to excretion (Enayati et al., 2005). GSTs are classified into two families based on their cellular location (microsomal and cytosolic). Within insects, cytosolic or canonical GSTs are divided into six classes (delta, sigma, epsilon, zeta, theta, and omega; Enayati et al., 2005; Ketterman et al., 2011). In the omega class, *Glutathione S transferase O3* ( *GstO3* ) is a biochemically active GST (Kim et al., 2006). It is significantly upregulated following exposure to oxidative stressors, such as hydrogen peroxide and zinc (Yepiskoposyan et al., 2006; Li et al., 2008). Across 12 *Drosophila* genomes, *GstO3* is highly conserved and expressed in all life stages of *D. melanogaster* (Walters et al., 2009).” (Williams et al., 2026)

We propose a gene model for the *D. immigrans* ortholog of the *D. melanogaster* *Glutathione S transferase O3* ( *GstO3* ) gene. The genomic region of the ortholog corresponds to the Augustus gene prediction JAECXE010002334 .g842.t1 in the ASM1815216v1 Genome Assembly of *D. immigrans* ( GCA_018152165.1 – Kim et al., 2021). This model is based on mixed sex adult fly RNA-Seq data from *D. immigrans* (Erlenbach et al., 2023; https://doi.org/10.5061/dryad.hdr7sqvq2 ) and *GstO3* in *D. melanogaster* using FlyBase release FB2024_02 ( GCA_000001215.4 ; Gramates et al., 2022; Jenkins et al., 2022; Larkin et al., 2021).

***Synteny***

The reference gene, *GstO3 ,* occurs on chromosome 3L in *D. melanogaster* and is flanked upstream by *CG6765* and *CG6683* and downstream by *sepia* ( *se* ) and *Glutathione S transferase O2* ( *GstO2* ). The *tblastn* search of *D. melanogaster* GstO3-PA (query) against the *D. immigrans* (GenBank Accession: GCA_018152165.1 Genome Assembly (database) placed the putative ortholog of *GstO3* within contig_2405 ( JAECXE010002334 ) which corresponds to Augustus gene prediction JAECXE010002334 .g842.t1 (E-value: 2e-130;percent identity: 71.37% as determined by *blastp* ). The putative ortholog is flanked upstream by Augustus gene prediction JAECXE010002334 .g843.t1 and JAECXE010002334 .g844.t1, which correspond to *CG6765* and *CG6683* in *D. melanogaster* (E-value: 0.0 and 8e-28; identity: 62.11% and 54.35%, respectively, as determined by *blastp* ; Figure 1A ; Altschul et al., 1990). The putative ortholog of *GstO3* is flanked downstream by Augustus gene prediction JAECXE010002334 .g841.t1 and JAECXE010002334 .g840.t1, which correspond to *se* and *GstO2* in *D. melanogaster* (E-value: 4e-166 and 3e-128; identity: 91.77% and 74.90%, respectively, as determined by *blastp* ). The putative ortholog assignment for *GstO3* in *D. immigrans* is supported by the following evidence: The gene predictions surrounding the *GstO3* ortholog are orthologous to the genes at the same locus in *D. melanogaster* , gene expression data corresponds with each prediction, and local synteny is completely conserved, supported by E-values and percent identities, so we conclude that Augustus gene prediction JAECXE010002334 .g842.t1 is an ortholog of *GstO3* in *D. immigrans* ( Figure 1A ).

***Protein Model***

*GstO3* in *D. immigrans* has 2 CDSs within the genome sequence. The only unique protein sequence (GstO3-PA) is translated from 1 mRNA isoform (GstO3-RA; Figure 1B ). Relative to the ortholog in *D. melanogaster* , the CDS number and protein isoform count are conserved (2 CDSs and 1 mRNA isoform *).* The sequence of GstO3-PA in *D. immigrans* has 71.4% identity (83.4% 85.1% similarity) with the protein-coding isoform GstO3-PA in *D. melanogaster* , as determined by *blastp* ( Figure 1C ). This level of divergence is not surprising given that *D. immigrans* and *D. melanogaster* belong to two separate subgenera ( *Drosophila* and *Sophophora* respectively) that diverged approximately 45-60MYA (Russo et al., 1995; Tamura et al., 2004; Obbard et al., 2012). Coordinates of this curated gene model are archived in the CaltechDATA repository (see “Extended Data” section below).

## Methods

The annotation methods used in this project are adapted from those described in Rele et al. (2023), which includes algorithms, database versions, and citations for the complete annotation process developed for the Pathways Project. The methods for the current project are detailed in brief below with notes on significant differences between this protocol and the one described in Rele et al. (2023). The students use the GEP instance of the UCSC Genome Browser v.435 ( https://gander.wustl.edu ; Kent et al., 2002; Raney et al., 2024) to examine the genomic neighborhood of their reference detoxification gene in the *D. melanogaster* genome assembly (Aug. 2014; BDGP Release 6 + ISO1 MT/dm6). Students obtain the protein sequence for the *D. melanogaster* target gene for a given isoform and use a *tblastn* search of the sequence against their target *Drosophila* species genome assembly ( *D. immigrans* ( GCA_018152165.1 – Kim et al., 2021)) on the NCBI BLAST server ( https://blast.ncbi.nlm.nih.gov/Blast.cgi , Altschul et al., 1990) to identify the putative ortholog location. Students compare the genomic neighborhood of the putative ortholog to that of the reference gene in *D. melanogaster* . This local synteny analysis includes a minimum of two upstream and downstream genes relative to the potential ortholog. As no RefSeq protein data is available for these species, comparisons are based on gene predictions that correlate with gene expression data in the putative ortholog neighborhood. Using the multiple alignment tracks feature in the Genome Browser, students examine other sets of genomic evidence, including Spaln alignment of *D. melanogaster* proteins, multiple gene prediction tracks (e.g., GeMoMa, Augustus, NSCAN PASA-EST), and RNA-Seq mixed sex adult fly expression data from the target species generated by Erlenbach et al. (2023; https://doi.org/10.5061/dryad.hdr7sqvq2 ). Information on the genomic structure information (e.g., CDSs, intron-exon number, number of isoforms) for the reference gene in *D. melanogaster* is retrieved using Gene Record Finder ( https://gander.wustl.edu/~wilson/dmelgenerecord/index.html ; Rele et al *.,* 2023). To determine approximate splice sites within the target gene, a *tblastn* search using the CDSs from the *D. melanogaste* r reference gene against the putative ortholog location (10kb up- and downstream of the target gene prediction). Coordinates of the CDS(s) are refined by examining aligned RNA-Seq data, identifying canonical splice site sequences, and ensuring the maintenance of an open reading frame. Students confirm the biological validity of their target gene model using the FlySeq Gene Model Checker ( https://gander2.wustl.edu/~wilson/genechecker-flyseq/ ), which compares the hypothesized target gene model's structure and translated sequence against the *D. melanogaster* reference gene. At least two independent models for this gene are generated. These models are reconciled by a third independent researcher to produce the final model presented here. Note: comparison of 5' and 3' UTR sequence information is not included in this GEP CURE protocol.

## Data Availability

Description: Zipped archive containing FASTA, PEP, and GFF. Resource Type: Dataset. DOI: https://doi.org/10.22002/w2mrj-8w313
